# Spectrum of Surgical Site Infection Pathogens in Chronic Infectious Spondylitis Requiring Revision Surgery: A 5-Year Cohort Study

**DOI:** 10.3390/jcm13061592

**Published:** 2024-03-11

**Authors:** Denis Naumov, Arkady Vishnevsky, Natalia Linkova, Dmitrii Medvedev, Alexander Krasichkov, Olga Sokolova, Victoria Polyakova, Piotr Yablonskiy

**Affiliations:** 1 St. Petersburg Research Institute of Phthisiopulmonology, 2–4 Ligovskii Ave., 191036 St. Petersburg, Russia; dg.naumov@spbniif.ru (D.N.); op.sokolova@spbniif.ru (O.S.);; 2 St. Petersburg Institute of Bioregulation and Gerontology, 3 Dynamo Ave., 197110 St. Petersburg, Russia; rsc-ide@yandex.ru; 3 Department of Radio Engineering Systems, Electrotechnical University LETI, 5F Prof. Popova St., 197022 St. Petersburg, Russia; 4 Department of Hospital Surgery, Faculty of Medicine, St. Petersburg University, 7–9 Universitetskaya Ave., 199034 St. Petersburg, Russia

**Keywords:** chronic infectious spondylitis, surgical site infection, cohort study

## Abstract

**Background:** Spectrum monitoring of the pathogen in spondylitis patients plays a key role in preventing infectious complications of spinal reconstructions in chronic spondylitis (CS) and in the treatment of surgical site infection (SSI). The aim of this study is to characterize the spectrum of SSI pathogens in CS requiring revision surgery. **Methods:** The primary cohort encompassed 569 surgical patients with infectious CS. In 99 patients (61 men and 38 women) requiring revision surgical interventions due to SSI, continuous microbiological monitoring of the pathogens was conducted. The average age of the patients was 63 ± 14 years. The vast majority of the patients underwent surgery on a set of multilevel (two or more spinal–motor segments) lesions. Lesions of the lumbar spine were more often noted, and lesions of the thoracic, thoracolumbar, and cervical spine sections were less often noted. This study included all patients operated on within the scope of revision spinal reconstructions in connection with the development of infection of the surgical area over the period from January 2018 to December 2022. Inclusion criteria were etiologically verified spondylitis, age of 18 years or older, and follow-up of 6 months or more. **Results:** The average rate of revision surgery due to SSI was 17.4%. Germ detection from the material of vertebral localization was noted in 48.3% and pathogen strains were isolated in urine in 60.8%, in decubital ulcers in 23.9%, and in hemoculture in 15.2% of all study patients. Aseptic, deep SSI was detected in 10.1%. Gram-positive, multidrug-resistant, and Gram-negative bacteria with extreme resistance prevailed in the microbiological landscape of late SSI, early, and delayed Gram-positive strains without drug resistance. **Conclusions:** Infectious etiology of spondylitis is associated with a significantly higher frequency of SSI. In the absence of a positive result from bacteriological examination of the vertebral localization material, it is advisable to conduct blood, decubital ulcer discharge, and urine sampling.

## 1. Introduction

Chronic infectious spondylitis (CS) is an etiologically heterogeneous group of destructive infections characterized by the anterior spinal column [1,2,3]. The standard treatment for acute CS forms (disease duration of no more than 30 days) includes isolated etiotropic antibacterial therapy for types A1–B2 according to Pola et al. (2017) or surgical sanitation of the infectious process area combined with extrafocal instrument fixation and lengthy etiotropic antibacterial therapy [4]. The most important stage of preoperative verification of spondylitis is percutaneous trepan biopsy from the vertebral lesion, followed by bacteriological, molecular genetic, and histological examination of the surgical material [5,6]. The frequency of verification of the pathogen through this method reaches 35–47% in acute infectious spondylitis, while the assignment of empirical antibacterial therapy at the first stage of treatment reduces the chances of detecting the pathogen microorganism to 14–21% [7,8,9].

When spondylitis becomes chronic (therapeutic pause lasts 3 months or more), not only is sanitation of the purulent focus necessary, but also three-column spinal reconstruction with correction of the sagittal balance parameters as a key criterion for ensuring the patient’s quality of life in the post-surgical period [10,11]. Isolated autosteal grafts have long remained the gold standard for the reconstruction of the anterior column of the spine. However, the high frequency of pseudoarthrosis, the progression of kyphotic deformity, and the development of spinal instability over the long term have dictated the need for the use of non-biological titanium mesh cages filled with auto-fluid for anterior fusion [12,13]. At the same time, the use of multi-support posterior instrumentation has ensured reliable correction of the spine’s sagittal profile and the preservation of the achieved parameters over the long term [14].

Despite the improvement of spinal instrumentation and technical instruments and the accumulation of surgical experience, such operations are associated with a high duration (from 5 to 7 h) and a significant volume of blood loss (from 800 mL to 2.1 L) [15,16]. Such operations are associated with high risks for developing deep surgical site infection (SSI) in the early (first 30 days after intervention) and late periods [17,18,19]. The use of various approaches is recommended depending on the timing of the postoperative complication. Thus, in conditions of early SSI in the field of surgical intervention stages, surgical site debridement, the imposition of negative-pressure wound therapy, and antibacterial therapy, taking into account the results of bacteriological research to preserve metal structures, are recommended. The development of delayed and late infection requires a more radical tactic—rehabilitation of the infection zone, the removal of implants, and a transition to non-focal osteosynthesis systems [20]. An important role is played by the localization of the infection, as the structure may be superficial or deep. The development of a deep infection in the field of surgical intervention requires the removal and replacement of metal structures as the main zone of the formation of microbial biofilms [21].

The progression of the infectious process often leads to the formation of two pathological conditions: loss of segmental stability with the development of pseudo-arthrosis and secondary vertebral canal stenosis with neurological disorders [22,23,24,25]. The main tasks to be solved during revision surgery include halting the signs of local and systemic inflammation, restoring vertebral stability, and decompressing intra-canal neural structures [26,27].

One of the causes of SSI is the high resistance of the microorganism strains to antibacterial drugs, which, according to different data, fluctuates from 1.1% to 7.4% [28,29,30]. Despite the optimized systems for perisurgical systemic antibacterial therapy in improved methods of SSI prevention (intra-wound use of vancomycin, irrigation of the surgical field with iodine-containing antiseptic solutions), the frequency of infectious complications developing after reconstructive surgery under chronic CS conditions reaches 21–29% [19,31,32].

The modern literature describes in detail the primary spinal reconstructions in chronic infectious spondylitis. At the same time, the specifics of conducting revision interventions, analyzing the microorganisms that cause infection in the surgical intervention area, and monitoring their drug resistance in chronic infectious spondylitis are poorly described.

Monitoring the SSI pathogen’s microbiological spectrum plays a key role in preventing infectious complications in primary reconstructions during chronic CS and the treatment of SSI in patients requiring revision interventions. 

The aim of this study is to characterize the spectrum of infection pathogens at the surgical intervention site in CS. To achieve this goal, we evaluated the pathogenic microorganism’s characteristics depending on the period of SSI development. We also characterized the pathogen’s drug resistance structure depending on the respective SSI development period.

## 2. Materials and Methods

The continuous monocentric cohort study, corresponding to class III, was conducted in the period from 10 January 2018 to 31 December 2022. All of the subjects gave their informed consent to inclusion before participating in the study. This study was conducted in accordance with the Declaration of Helsinki, and the protocol was approved by the Ethics Committee of the St. Petersburg Research Institute of Phthisiopulmonology (Project identification code—No. 358; date: 6 June 2018).

Patients were included in the study on the basis of the following criteria: CS at the time of the primary reconstructive surgery, the presence of SSI requiring revision surgery, a patient age of 18 years or older at the time of revision surgery, verification of wound cavity discharge with determination of the SSI pathogen drug resistance spectrum, verification of the pathogen from hemoculture (triple sampling at the height of fever), urine, subclavian catheters, and decubital ulcer superficial discharge during SSI, and follow-up tracked for 6 months or more. The exclusion criterion was no indications for revision surgical intervention.

During the analyzed period, 569 patients with chronic CS underwent surgery. Of these patients, 364 (63.9%) underwent primary reconstructions and 205 (36.1%) underwent surgery for revision surgical interventions (primary surgery was performed outside of the St. Petersburg Research Institute of Phthisiopulmonology). The final cohort consisted of 99 patients who, at different intervals during the postoperative period, had recorded SSI requiring repeat surgery. The study included all patients operated on within the scope of revision spinal reconstructions in connection with the development of infection of the surgical area. Inclusion criteria were etiologically verified spondylitis, age of 18 years or older, and follow-up of 6 months or more.

The data of 61 male and 38 female patients were analyzed. The average age of the patients at the time of revision surgery was 63 ± 14 years old. The assessment of patients’ comorbidity was conducted using the Charlson index, which, in the study cohort, was 7.4 ± 1.2 points. Diabetes mellitus (32 patients, 32.3%), chronic viral hepatitis C (17 patients, 17.1%), and BMI ≥ 30 (12 patients, 12.1%) prevailed in the structure of predictors of the development of the surgical area’s infection.

The vast majority of patients (74 cases) underwent surgery on a set of multilevel (2 or more spinal–motor segments) lesions. Lesions of the lumbar spine (56 cases) were more often noted, while lesions of the thoracic (23 cases), thoracolumbar (12 cases), and cervical (8 cases) spine sections were noted less often. The general characteristics of the patients included in the study are presented in Table 1.

Trepan biopsy under X-ray control was performed in all cases of deep infection of the surgical area at the stage of preparation for revision surgery. The criteria for the diagnosis of “infection in the area of surgical intervention” were visible inflammatory changes in soft tissues in the area of surgical intervention and the presence of a fistula communicating with the area of surgery. Superficial infection was limited to subcutaneous fat and muscle fascia. Deep infection was diagnosed in the event of communication with a metal structure. The development of the infectious process was also characterized by an increased level of Leu, ESR, and CRP proinflammatory markers in the blood and a 3-fold positive result of a bacteriological blood test against the backdrop of fever. Signs of infectious changes in the anterior or posterior fusion zone were revealed according to computed tomography and magnetic resonance imaging data: pre-/paravertebral, epidural abscesses, peri-implant bone resorption, and Modic I type changes.

Bacteriological examination of the wound discharge was performed in all cases at the stage of preparation for revision surgery. The material was obtained through aspiration of the wound with superficial infection of the surgical area and with X-ray-navigated biopsy in the case of deep infection of the surgical area. Closed trepan biopsy was performed from the lesion under the control of an electron-optical converter during the establishment of the diagnosis “deep infection of the surgical intervention area”. Trepan biopsy was performed by passing a Jamshidi needle along a transpedicular trajectory in the area of the cranial and caudal blocked segments (spongy bone tissue samples were taken), as well as through the Kambin triangle to take material from the intervertebral disc. Aspiration of the wound and the contents of the fistula was performed in all cases of a superficial infection of the surgical area. The obtained biological material was sent for bacteriological, molecular genetic, and histological (bone fragment) studies.

Bacteriological blood sterility testing was performed three times in all patients. The scope of the preoperative radiation examination included MRI and CT scans of the affected spine. Empirical antibacterial therapy was performed in 10 patients (10.1%) with negative results of the bacteriological examination of wound discharge, as well as in the case of negative blood and urine cultures.

**Figure 1:**
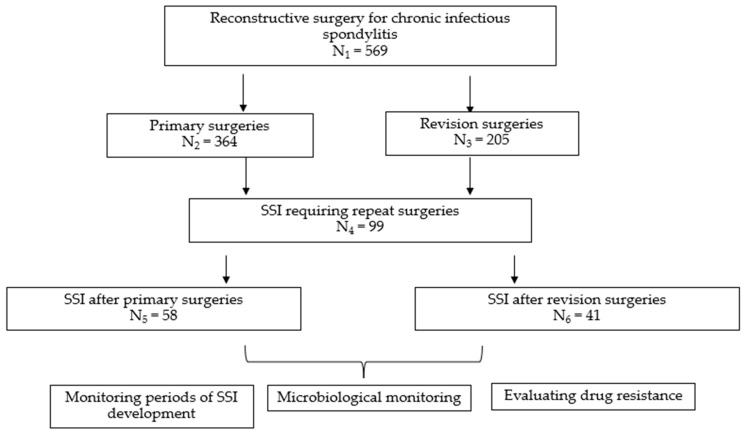
Study plan.

The study plan is depicted in Figure 1.

The following parameters were analyzed: type of SSI microorganism–pathogen, SSI microorganism–pathogen’s drug resistance spectrum, and SSI development period according to the division into periods per Prinz V. et al. 2020 (early ≤ 6 weeks, delayed > 6 weeks, late > 12 months) [20]. Biological material was collected for bacteriological study before revision surgery from the wound discharge (if there was a fistulous process) and directly during the intervention (granulation, pus). If delayed and late SSI and an unstable rear metal structure were detected during removal of the support components, the implant’s surfaces were given ultrasound treatment with subsequent bacteriological study.

Nonspecific microflora and *Mycobacterium* spp. were identified on the basis of inoculations on dense and liquid nutrient media, DNA detection of the mycobacterium tuberculosis complex, and amplification of the nucleotide sequence IS6110; a marker of the mycobacterium tuberculosis complex was conducted using the test system SPE DNA-Technology (DNA Technology, Moscow, Russia) through the PCR method in real time on the iCyclerQ, Bio-Rad analyzer (Bio-Rad Laboratories, Inc., Hercules, CA, USA). The threshold value for the colony-forming unit (CFU) of SSI microorganism–pathogens for inclusion in the study was adopted as ≥10^5^ (a lower CFU value was considered a version of sample contamination). In all instances, the bacteriological study of biomaterial for anaerobic and facultative–anaerobic microorganisms was supplemented by determination of the drug sensitivity of the SSI pathogens using the disc-diffusion method based on EUCAST (2020) recommendations. According to the recommendations from the Clinical Laboratory Standards Institute (CLSI), the European Committee on Antimicrobial Susceptibility Testing (EUCAST), and the US Food and Drug Administration (FDA), the structure of drug susceptibility revealed microorganisms with multi-resistance (resistance to one antibacterial drug in three or more drug groups), extreme resistance (resistance to one or more antibacterial drugs in all groups, with the exception of categories 1–2), and pan-resistance (resistance to all antibacterial drugs in all groups) [33]. A histological study was conducted based on an analysis of vertebral localization material obtained through paracentetic trepanobioposy.

A statistical analysis was conducted based on recommendations from the World Osteosynthesis Association and Falavigna A. et al. (2015) [34]. The software Statistical Package for the Social Sciences (SPSS), version 22.0 (SPSS Inc., Chicago, IL, USA) was used. The studied parameters were checked for normal distribution using the Kolmogorov–Smirnov criterion. The Kruskal–Wallis N-criterion was used to evaluate the significance of the differences in SSI development periods. The effect of spondylitis etiology on the drug sensitivity spectrum was verified using Pearson’s x^2^ criterion with the construction of conjugation tables. The differences were considered statistically reliable with two-sided *p* ˂ 0.05.

## 3. Results

The greatest frequency in complications was noted in the late postoperative period, with 54 observations (54.5%); they were recorded less often in the early (31 patients) and delayed (14 patients) periods (x^2^ = 9.237, *p* = 0.009). SSI pathogens were identified in 89 patients (89.8%); in this case, the microorganism was isolated from the vertebral localization material in 43 cases (48.3%). In cases of “culture negative” SSI, combined with clinical manifestations (fistulous process in the surgical area, elevated C-reactive protein level > 10 mg/L, and ESR > 30 mm/h) and with histological signs of inflammation, pathogen strains were isolated in the urine in 28 (60.8%), in decubital ulcers in 11 (23.9%), and in hemoculture in 7 (15.2%) of the patients, respectively. For 10 patients (10.1%) with clinical SSI signs, histological inflammation signs, and a negative result from the bacteriological material study of both vertebral and other localization, an SSI diagnosis was established with an unidentified pathogen; here, in all cases, it was identified in the late, postoperative period. Chronic spondylitis etiology had a significant impact on the frequency of SSI development; thus, postoperative complications under conditions of chronic non-specific spondylitis were noted in 67 cases (67.6%), while during tuberculous spondylitis they were noted in 32 (32.3%) of the cases (x^2^ = 21.345, *p* < 0.001). Analysis of the microorganism types revealed significant differences. Multi-resistant Gram-positive and Gram-negative bacteria with extreme resistance were found more often in patients with late SSI, while pathogens of early and delayed SSI were more often Gram-positive bacteria without drug resistance (x^2^ = 17.516, *p* = 0.0032). Two microorganisms were verified in 32 patients, and three were verified in 6 people.

The distribution of the detected vertebral localization isolates, depending on the SSI development period, is shown in Table 2.

In the evaluation of the drug resistance of pathogens obtained from vertebral localization, the specific weight of Gram-positive multi-resistant strains was 30.3%. Among them, *Staph. epidermidis* (MRSE) accounted for 63% and *Staph. aureus* (MRSA) accounted for 37%. The percentage of stable strains in the Gram-positive bacteria reached 80%. Among them, 87.5% of the isolates had extreme resistance (*Klebsiella* spp. and *Acinetobacter* spp.), while 12.5% had pan-resistance (*Pseud. aureginosa*).

## 4. Discussion

Revision surgery with spondylitis is one of the most complex challenges in spinal surgery, insofar as it is accompanied by a large number of infectious complications and the high economic costs associated with the rendering of specialized medical care [35,36]. Despite the increased number of publications in this field, there is virtually no information about the late results of revision interventions in patients with chronic CS, while data reflecting the results of microbiological monitoring in this patient cohort are only found in a few publications [1,4,16,17].

While predicting SSI development in spinal surgery is still relevant, a consensus opinion on the subject remains elusive. The risk factors for SSI development are both patient-associated (Charlson index, body mass index, comorbid rheumatological pathology, and others) and intervention-associated (primary/revision surgery, its duration, blood loss, and more) [19,31,37]. Mueller K. B. et al. (2022) suggested an SSI development risk scale after vertebral interventions that views the revisional nature of the surgery as one of the most significant predictors of infectious complications [22].

A study of late results in patients undergoing surgery for non-specific spondylodiscitis demonstrates the significant effect of the microorganism–pathogen type on mortality. According to Kehrer M. et al. (2015), mortality in the early and delayed post-surgical period is higher in patients with multi-resistant strains of *Staph. aureus*, while in the late period, muti-resistant Gram-positive microorganisms were found more often [36]. Our study identified similar results: one fatal outcome was recorded in patients with late, deep SSI associated with Gram-negative extremely resistant bacteria.

One of the methods for preventing SSI during initially “sterile” operations on the spine is the intra-wound use of vancomycin. A meta-analysis by Shan S. et al. (2020) indicates the significant effect of this method on the risk of developing infectious complications [38]. The nature of the microorganisms associated with SSI in the early post-surgical period is also noteworthy. Among them, 75% of the cases identified Gram-positive bacteria, which is a recommendation for the intra-wound use of vancomycin in this patient category as well.

In the general cohort of SSI pathogens, the main strains were Gram-positive microorganisms identified in 76.7% of the cases. The leader among them is *Staphylococcus* spp. Representatives from *Enteococcus* spp., predominantly *E. faecalis* and *E. faecium*, were found in the nosocomial flora structure; here, the pathogen was detected in the urine and decubital ulcer discharge. Multi-resistance to fluoroquinolones of the third and fourth generations was a feature of this microorganism group. Similar trends were detected during 6-year monitoring of the structure and resistance of the leading pathogens [39].

## 5. Conclusions

Microbiological monitoring of patients with chronic CS revealed the following. There is a predominance in the structure of SSI pathogens of Gram-positive bacteria with drug resistance. The development of late SSI is due to multi-resistant Gram-positive and extremely resistant Gram-negative microorganisms. The non-specific etiology of spondylitis is linked to the high development frequency of SSI. In the absence of a positive result from the bacteriological study of vertebral localization material, it is expedient to take a blood, decubital ulcer discharge, and urine sampling. The use of the obtained data will optimize the schemes of perioperative antibacterial prevention and help reduce the incidence of infection in the field of surgical intervention in patients with chronic infectious spondylitis.

## Figures and Tables

**Table 1:** General characteristics of the patients.

Gender	Age	Localization/Extent of Destruction
Men, 61 patients; Women, 38 patients	63 ± 14 years	Polysigmental—74 patients; Monosigmental—25 patients; C (neck)—8 cases; Th (thoracic)—23 cases; Th/L (thoracolumbar)—12 cases; L (lumbar)—56 cases

**Table 2:** Presence of a certain type of microorganism, depending on the SSI development period.

	Total (abs. and %)	Early SSI (abs. and %)	Delayed SSI (abs. and %)	Late SSI (abs. and %)
Gram (+):	33/76.7%	8/18.6%	4/9.3%	11/25.5%
Including multi-resistant	10/30.3%	1/2.3%	1/2.3%	8/18.6% *
Gram (−):	10/23.2%	2/20%	1/10%	–
Including extremely resistant	6/60%	1/10%	1/10%	4/40% *
Pan-resistant	1/10%	–	–	1/10%

* Statistically significant differences were found across the groups of early, delayed, and late SSI using the Kruskal–Wallis N-criterion, x^2^ = 17.516, *p* = 0.0032. abs.—absolute value.

## Data Availability

Data are contained within the article.

## References

[1] Lora-Tamayo J., Euba G., Narváez J.A., Murillo O., Verdaguer R., Sobrino B., Narváez J., Nolla J.M., Ariza J. (2011). Changing trends in the epidemiology of pyogenic vertebral osteomyelitis: The impact of cases with no microbiologic diagnosis. Semin. Arthritis Rheum..

[2] Stricsek G., Iorio J., Mosley Y., Prasad S., Heller J., Jallo J., Shahrokh S., Harrop J.S. (2018). Etiology and surgical management of cervical spinal epidural abscess (SEA): A systematic review. Glob. Spine J..

[3] Yoon S.H., Chung S.K., Kim K.J., Kim H.J., Jin Y.J., Kim H.B. (2010). Pyogenic vertebral osteomyelitis: Identification of microorganism and laboratory markers used to predict clinical outcome. Eur. Spine J..

[4] Pola E., Autore G., Formica V.M., Pambianco V., Colangelo D., Cauda R., Fantoni M. (2017). New classification for the treatment of pyogenic spondylodiscitis: Validation study on a population of 250 patients with a follow-up of 2 years. Eur. Spine J..

[5] Yunoki M. (2023). Comprehensive review of pyogenic spondylitis management for neurosurgeons. Asian J. Neurosurg..

[6] Giampaolini N., Berdini M., Rotini M., Palmisani R., Specchia N., Martiniani M. (2022). Non-specific spondylodiscitis: A new perspective for surgical treatment. Eur. Spine J..

[7] Dai G., Li S., Yin C., Sun Y., Hou J., Luan L., Liu C., Wang Z., Cao Z., Wang T. (2021). Culture-negative versus culture-positive in pyogenic spondylitis and analysis of risk factors for relapse. Br. J. Neurosurg..

[8] Cui Y., Mi C., Wang B., Zheng B., Sun L., Pan Y., Lin Y., Shi X. (2022). Manual homogenization improves the sensitivity of microbiological culture for patients with pyogenic spondylitis. Infect. Drug Resist..

[9] Kim N.J. (2021). Microbiologic Diagnosis of Pyogenic Spondylitis. Infect. Chemother..

[10] Bazarov A., Naumov D., Mushkin A., Sergeyev K., Ryabykh S., Vishnevsky A., Burtsev A., Mushkin M. (2022). A new classification of spondylodiscitis: Possibility of validation and multidisciplinary expert consensus. Spine Surg..

[11] Abboud T., Krolikowska-Fluori M., Melich P., Rohde V., Schatlo B. (2023). Postoperative quality of life in patients with pyogenic spondylodiscitis. J. Neurol. Surg. A Cent. Eur. Neurosurg..

[12] Zhang H.Q., Wang Y.X., Guo C.F., Tang M.X., Liu S.H., Deng A., Gao Q. (2022). Posterior-only debridement, bone fusion, single-segment versus short-segment instrumentation for mono-segmental lumbar or lumbosacral pyogenic vertebral osteomyelitis: Minimum five year follow-up outcomes. J. Orthop. Surg. Res..

[13] Wu S., Lin B., Li X., Chen S., Zhang H., Wu Z., Tang S., Yang Y., Liang B. (2021). Single-stage debridement via autogenous iliac bone graft through the OLIF corridor and lateral fixation in treating spontaneous single-level lumbar pyogenic spondylodiscitis. BMC Musculoskelet. Disord..

[14] Zhang C.H., Zaidman N., Russo V. (2020). Hybrid minimally invasive technique for treatment of thoracolumbar spondylodiscitis and vertebral osteomyelitis. World Neurosurg..

[15] Inoue T., Kobayashi N., Baba N., Ide M., Higashi T., Inaba Y. (2023). Predictors of conversion surgery after conservative treatment for pyogenic spondylitis. J. Orthop. Sci..

[16] Kramer A., Thavarajasingam S., Neuhoff J., Ponniah H., Ramsay D., Demetriades A., Davies B., Shiban E., Ringel F. (2023). Epidemiological trends of pyogenic spondylodiscitis in Germany: An EANS Spine Section Study. Sci. Rep..

[17] D’Agostino C., Scorzolini L., Massetti A.P., Carnevalini M., d’Ettorre G., Venditti M., Vullo V., Orsi G.B. (2010). A seven-year prospective study on spondylodiscitis: Epidemiological and microbiological features. Infection.

[18] Wang X., Lin Y., Yao W., Zhang A., Gao L., Feng F. (2023). Surgical site infection in spinal surgery: A bibliometric analysis. J. Orthop. Surg. Res..

[19] Anderson P.A., Savage J.W., Vaccaro A.R., Radcliff K., Arnold P.M., Lawrence B.D., Shamji M.F. (2017). Prevention of surgical site infection in spine surgery. Neurosurgery.

[20] Prinz V., Vajkoczy P. (2020). Surgical revision strategies for postoperative spinal implant infections (PSII). J. Spine Surg..

[21] Tsiskarashvili A.V., Gorbatyuk D.S., Melikova R.E., Pkhakadze T.Y., Kazmin A.I., Suleimanov M.A. (2022). Microbiological spectrum of causative agents of implant-associated infection in the treatment of complications of transpedicular fixation of the spine using the negative pressure method. Russ. J. Spine Surg..

[22] Mueller K.B., Hou Y., Beach K., Griffin L.P. (2022). Development and validation of a point-of-care clinical risk score to predict surgical site infection following open spinal fusion. N. Am. Spine Soc. J..

[23] Park J., Han S., Jeon Y., Hong J.Y. (2023). Spinal epidural abscess as predicting factor for the necessity of early surgical intervention in patients with pyogenic spondylitis. BMC Musculoskelet. Disord..

[24] Naumov D.G., Tkach S.G., Mushkin A.Y., Makogonova M.E. (2021). Chronic infectious lesions of the cervical spine in adults: Monocentric cohort analysis and literature review. Spine Surg..

[25] Mushkin A.Y., Naumov D.G., Evseev V.A. (2019). Multilevel spinal reconstruction in pediatric patients under 4 years old with non-congenital pathology (10-year single-center cohort study). Eur. Spine J..

[26] Hu X., Lieberman I.H. (2018). Revision spine surgery in patients without clinical signs of infection: How often are there occult infections in removed hardware?. Eur. Spine J..

[27] Mengis-Palleck C.L., Tomé-Bermejo F., Piñera-Parrilla Á., Cervera-Irimia J., Gallego-Bustos J., Garzón-Márquez F., Rodríguez-Arguisjuela M.G., Sanz-Aguilera S., Peiro-Garcia A., Álvarez-Galovich L. (2023). Surgical site infection after polymethyl methacrylate pedicle screw augmentation in osteoporotic spinal vertebrae: A Series of 537 cases. Int. J. Spine Surg..

[28] Kang S.-J., Jang H.-C., Jung S.-I., Choe P.G., Park W.B., Kim C.-J., Kim H.B., Oh M.D., Kim N.J., Park K.H. (2015). Clinical characteristics and risk factors of pyogenic spondylitis caused by Gram-negative bacteria. PLoS ONE.

[29] Zhou J., Wang R., Huo X., Xiong W., Kang L., Xue Y. (2020). Incidence of surgical site infection after spine surgery: A systematic review and meta-analysis. Spine.

[30] Prinz V., Bayerl S., Renz N., Trampuz A., Czabanka M., Woitzik J., Vajkoczy P., Finger T. (2019). High frequency of low-virulent microorganisms detected by sonication of pedicle screws: A potential cause for implant failure. J. Neurosurg. Spine.

[31] Shirai T., Tsuchiya H., Terauchi R., Tsuchida S., Shimomura S., Kajino Y., Takahashi K. (2023). Iodine-supported implants in prevention and treatment of surgical site infections for compromised hosts: A prospective study. J. Orthop. Surg. Res..

[32] Agarwal A., Lin B., Wang J.C., Schultz C., Garfin S.R., Goel V.K., Anand N., Agarwal A.K. (2019). Efficacy of ontraoperative implant prophylaxis in reducing intraoperative microbial contamination. Glob. Spine J..

[33] Magiorakos A.P., Srinivasan A., Carey R.B., Carmeli Y., Falagas M.E. (2012). Multidrug-resistant, extensively drug-resistant and pandrug-resistant bacteria: An international expert proposal for interim standard definitions for acquired resistance. Clin. Microbiol. Infect..

[34] Falavigna A., Jiménez Avila J.M. (2015). Education in Research: From the Idea to the Publication.

[35] Blumberg T.J., Woelber E., Bellabarba C., Bransford R., Spina N. (2018). Predictors of increased cost and length of stay in the treatment of postoperative spine surgical site infection. Spine J..

[36] Kehrer M., Pedersen C., Jensen T.G., Hallas J., Lassen A.T. (2015). Increased short- and long-term mortality among patients with infectious spondylodiscitis compared with a reference population. Spine J..

[37] Abdallah D.Y., Jadaan M.M., McCabe J.P. (2013). Body mass index and risk of surgical site infection following spine surgery: A meta-analysis. Eur. Spine J..

[38] Shan S., Tu L., Gu W., Aikenmu K., Zhao J. (2020). A meta-analysis of the local application of vancomycin powder to prevent surgical site infection after spinal surgeries. J. Int. Med. Res..

[39] Bozhkova S.A., Kasimova A.R., Tikhilov R.M., Polyakova E.M., Rukina A.N., Shabanova V.V., Liventsov V.N. (2018). Adverse Trends in the Etiology of Orthopedic Infection: Results of 6-Year Monitoring of the Structure and Resistance of Leading Pathogens. Traumatol. Orthop. Russ..

